# On the existence of soliton-like collective modes in liquid water at the viscoelastic crossover

**DOI:** 10.1038/s41598-021-84277-8

**Published:** 2021-03-08

**Authors:** V. E. Zakhvataev, L. A. Kompaniets

**Affiliations:** 1 Federal Research Center “Krasnoyarsk Scientific Center of the Siberian Branch of the Russian Academy of Sciences”, 660036 Krasnoyarsk, Russia; 2 Siberian Federal University, 660041 Krasnoyarsk, Russia; 3 Institute of Computational Modelling of the Siberian Branch of the Russian Academy of Sciences, 660036 Krasnoyarsk, Russia

**Keywords:** Chemical physics, Structure of solids and liquids

## Abstract

The problem of large-density variations in supercooled and ambient water has been widely discussed in the past years. Recent studies have indicated the possibility of nanometer-sized density variations on the subpicosecond and picosecond time scales. The nature of fluctuating density heterogeneities remains a highly debated issue. In the present work, we address the problem of possible association of such density variations with the dynamics of terahertz longitudinal acoustic-like modes in liquid water. Our study is based on the fact that the subpicosecond dynamics of liquid water are essentially governed by the structural relaxation. Using a mode coupling theory approach, we found that for typical values of parameters of liquid water, the dynamic mechanism coming from the combination of the structural relaxation process and the finiteness of the amplitude of terahertz longitudinal acoustic-like mode gives rise to a soliton-like collective mode on a temperature-dependent nanometer length scale. The characteristics of this mode are consistent with the estimates of the amplitudes and temperature-dependent correlation lengths of density fluctuations in liquid water obtained in experiments and simulations. Thus, the fully dynamic mechanism could contribute to the formation and dynamics of fluctuating density heterogeneities. The soliton-like collective excitations suggested by our analysis may be relevant to different phenomena connected with supercooled water and can be expected to be associated with some ultrafast biological processes.

## Introduction

The existence of density variations in both supercooled and ambient water on the subnanometer/nanometer length scales and subpicosecond/picosecond time scales has been widely discussed in recent years^1–29^. Bulk water under ambient conditions is traditionally considered in the framework of a homogeneous model as a mainly tetrahedrally hydrogen-bonded, continuous liquid^2–5,7–9,13,14,21,22,30,31^. Within this model, the temporal existence of low- and high-density regions in liquid water can be explained as a result of natural density fluctuations of an equilibrium system. For instance, molecular dynamics (MD) simulations based on the SPC/E model showed the presence of transient low- and high-density (< 0.9 or > 1.1 kg/m^3^) regions in water for both supercooled and ambient conditions, interpreted within a continuum model. The correlation length of the corresponding density fluctuations was estimated to be of 0.7 nm, and the timescale for low- and high-density regions was estimated as < 4 ps^8^. Likewise, numerical simulations by Soper^3^ showed that on the length scale of 0.9 nm, density fluctuations can, in principle, be up to ~  ± 30% compared to the average water density.

 Different local and global order parameters have been used in order to assess the presence of low- and high-density regions in liquid water^26,27^. Recently, coexisting low- and high-density regions in ambient water have been identified using Voronoi voids on a larger length scale of 1–2 nm than those predicted by local order parameters and those reported previously^19^. Atomistic simulations showed that the low- and high-density regions are formed around the regions of empty space within the hydrogen-bond network, occurring as a result of thermal fluctuations^18^. The regions of empty space are frequently formed as asymmetric, fractal voids that are highly delocalized over the hydrogen-bond network, and the corresponding nanometer-sized density fluctuations can be associated with these voids. The results of simulations^18^ suggested a picosecond time scale for voids with volumes larger than 0.15 nm^3^, while small voids can be characterized by a subpicosecond timescale. Similar results were obtained for supercooled water^29^.

 Large-density fluctuations in liquid water can be explained also in the framework of heterogeneous models based on fluctuations between two classes of different structures^12^, known as low-density liquid (LDL) and high-density liquid (HDL) structures^1^, locally favoured and normal structures^32^, as well as symmetrical and asymmetrical structures^33^. The LDL structure corresponds to tetrahedral local coordination, while the HDL structure is characterized by distorted first hydration shell and weakened or broken hydrogen bonds. The local environment of the LDL-like (HDL-like) species has, respectively, lower (higher) density than the average. An expected density difference between LDL-like and HDL-like species is 20–30%^21,34–36^, while an average length scale of density fluctuations was estimated as ~ 1.5 nm for ambient water^1,16^.

 Heterogeneous picture of ambient water was criticized by different authors^2–5,7–9,13,14,21,22,30,31^. This criticism was answered^6,12,23–25,37,38^. In particular, Duboue-Dijon and Laage^13^ studied a broad range of local order parameters for liquid water on the basis of MD simulations of bulk water and hydration shell of a hydrophobic solute, for both supercooled and ambient conditions. They include into consideration such widely used order parameters as the local structure index (LSI)^39^ and orientational tetrahedral order parameter. None of the distributions for the local order parameters considered showed any sign of a heterogeneous mixture. However, as Pettersson pointed out^24^, modern MD simulations seem to underestimate the magnitude and specificity of the fluctuations suggested by the experimental data. If one removes effects of thermal excitations considering the level of the inherent structure (the inherent structure is obtained by quenching the instantaneous structure into the nearest local minimum on the underlying potential energy surface of the simulation^24^), the distribution of the LSI becomes bimodal^40^, while without quenching to the inherent structure the LSI distribution is unimodal for most simulations of ambient water^21^. But in a very recent study^26^ accounting for the presence of thermal noise, the LSI distribution was nevertheless found to be bimodal.

 The existence of a bimodal aggregation of molecules with disordered and tetrahedral local environments is strongly supported by experimental data. Optical Kerr effect measurements of low-frequency modes^11^, studies using X-ray photon correlation^41^, X-ray absorption^42^, and X-ray emission spectroscopies^1,43^, infrared spectra^44^, as well as the full temperature- and polarization-dependent Raman spectra^45^ indicate the bimodal distribution in terms of LDL and HDL. Studies of the oxygen–oxygen pair distribution function of water provide further support to the heterogeneous models^28,46–48^.

 There is another argument. According to the heterogeneous models, two different local structural arrangements are related to high-density and low-density forms of liquid water postulated in the liquid–liquid transition hypothesis. More specifically, they are related to macroscopic liquid phases (pure low-density liquid and high-density liquid phases) with coexistence line between them, ending in a critical point in a metastable supercooled region^49^. Very recently, the LDL and HDL macroscopic liquid phases and the transition between them have been observed using wide-angle X-ray scattering and X-ray photon correlation spectroscopy upon heating of high-density amorphous ice into the ultraviscous regime^41^. Pure LDL has also been experimentally shown for the case of rapid decompression of ice VIII^50^. Also, a Fourier transform infrared spectroscopy gives an evidence for the existence of the LDL phase in supercooled confined water^51^. In addition, X-ray scattering at the low momentum transfer region on micrometer-sized water droplets cooled down to 227 K in vacuum points to the existence of a Widom line extending from a critical point in a liquid–liquid transition^16^. These findings are in agreement with theoretical estimates based on the data on hypersound propagation^52^. The liquid–liquid coexistence has also been reported for some molecular water models^12,15^. In particular, HDL and LDL coexistence as metastable phases at the thermodynamic conditions corresponding to a liquid–liquid transition has been shown in simulations of the ST2 model^53^. Experiments and simulations on nano-confined water^54^ and on protein hydration water^55,56^ provide further support to the liquid–liquid transition hypothesis. The fluctuations between HDL and LDL local structures appearing in the transition between the two phases has been proven to be fully consistent with thermodynamics^20,32,57^.

 It should be noted, however, that fluctuating regions of LDL and HDL structures in the framework of heterogeneous models of water do not represent metastable macroscopic phases, as opposed to LDL and HDL macroscopic phases at extreme conditions of supercooling and pressure^12^.

 At the same time, supercooled water is known to exhibit dynamical heterogeneity^58–60^, and heterogeneous models have been widely applied to describe water in the supercooled regime^61,62^. Heterogeneous models may also be relevant in confinement, at interfaces, and in solution^63,64^.

 Importantly, recent studies^27,65^ have shown that formation of nanometer-sized density variations can greatly influence the dynamics of liquid water on subpicosecond and picosecond time scales. Using MD simulations of TIP4P/2005 water, Camisasca et al.^27^ investigated the self- and distinct-van Hove functions for different local environments of water, classified using the initial value of the LSI of water molecules. The self-van Hove function determines autocorrelations of the individual particle motion, while the distinct-van Hove function describes the system rearrangement with respect to a fixed origin, initially occupied by a particle^27^. The LSI is a measure of local translational order which reflects the degree of order in the first and second coordination shells^27,39^. Camisasca et al.^27^ focused on the very extremes of the LSI distribution corresponding to two initial local structures, LDL-like and HDL-like structures. Simulations showed that a strong LDL (HDL) molecule enhances the LDL (HDL) character of the surrounding molecules, which in turn enhances the probability of the selected molecule to return to its original character after transient excursions to HDL (LDL)^27^. Thus although individual molecules do not persist in the same LDL or HDL population at all times, molecules are brought back by the preference of the surrounding molecules toward the initial arrangement^27^. The molecular arrangement around the LDL and HDL species was shown^27^ to be well-defined over time at the distances of the order of 2.2 nm. Notably, it was found that the initial structure of local environments plays an important role for the subsequent translational dynamics on a nanometer length scale and picosecond time scale, much longer than typically considered. It was shown that the translational dynamics of water molecules depend on the initial local structure around the molecule for a wide temperature range of 230–340 K^27^. The observed discrepancies in the self-van Hove functions calculated for different groups of molecules, based on their LSI value, indicate the heterogeneous translational dynamics of water, related to the existence of middle-range order^27^.

 In the simulations^27^ the effects connected with LDL-like and HDL-like species were quite local since a significant part of water molecules was sufficiently close to the average; thus the observed LDL-like and HDL-like species represented rather extreme and rare fluctuations. However, Iwashita et al.^17^ showed that the local molecular dynamics of ambient water can possibly reflect local fluctuations into LDL^17^. This conclusion is based on the results of direct experimental measurements of real-space, real-time motions of water molecules via the van Hove function determined by inelastic X-ray scattering. Iwashita et al.^17^ showed that water molecules are strongly correlated in space and time with coupling between the first and second nearest-neighbour molecules. Water seems to be different from some other liquids in this respect^17^. The observed local dynamics were found to be crucial to a fundamental understanding of the origin of the physical properties of water, including viscosity and molecular transport^17^.

 Starting structures classified by the LSI, seeding in the simulations^27^, may be treated as both extreme, rare spontaneous fluctuations or externally induced perturbations. As described above, these provide formation of LDL-like and HDL-like regions that are well-defined on nanometer length and picosecond time scales and strongly influence the translational dynamics of water^27^. Such density variations may be relevant in various cases, such as dynamical heterogeneity in supercooled water, a phenomenon where spatially separated, extended regions of relatively mobile and immobile molecules coexist^66^. Specifically, recent simulations showed that in supercooled water low-mobility regions have a lower local density (and higher degree of tetrahedrality), while high-mobility regions have a higher local density^61,62^. This finding has significant implications for the understanding of diverse phenomena associated with supercooled water; for example, it was shown that ice nucleation occurs in the low-density regions^67^.

 Large-density variations may also be relevant in the following context. Inelastic neutron scattering measurements allow to estimate that density fluctuations in biomolecular hydration water can be affected by the biomolecule surface at a distance up to 1.2 nm from the surface^68^. The results of THz spectroscopy suggest that this distance may be significantly larger. It was found that the perturbation induced by some proteins to the collective dynamics of hydration water can extend to about 2 nm or more from the protein-water interface^69^. Also, MD simulations showed the similarity of the acoustic-like modes in the protein and protein hydration water^64,70^. Such long-range protein-water interactions associated with heterogeneous hydration dynamics were proven to be able to contribute to the activity of proteins^64^. The possibility of similar long-range interactions between bilayer lipid membranes and solvent in the terahertz frequency range was indicated in Ref.^71^.

 Large-density variations can also arise in such natural processes as vibrations of nanoparticles in water in the terahertz frequency range which produce large-amplitude perturbations of adjacent water, regions of rarefication^72,73^. We finally note that recent simulations revealed the existence of propagating nanometer-sized (~ 5 nm) density pulses in liquid water, induced by a large-amplitude initial perturbation^74^.

 In the present work, we address the problem of possible association of the discussed nanometer-sized density variations with the dynamics of terahertz longitudinal acoustic-like modes in liquid water. Our analysis is based on the fact that the subpicosecond dynamics of liquid water are essentially governed by a (α-) structural relaxation process, i.e., the time decay of density fluctuations induced by viscous dissipation. Within the viscoelastic crossover region, the structural relaxation causes a positive sound dispersion, i.e., upward bending of the wave number-dispersion of the longitudinal acoustic-like mode from the low-frequency sound dispersion to the high-frequency dispersion^75–81^. Using a mode coupling theory approach, we show that for typical values of parameters of both bulk water and protein/DNA hydration water, the dynamic mechanism coming from the combination of the structural relaxation process and the finiteness of the amplitude of terahertz longitudinal acoustic-like mode gives rise to the existence of soliton-like collective modes on a temperature-dependent nanometer length scale. We also show that the characteristics of these modes are consistent with the estimates of the amplitudes and temperature-dependent length scales of density fluctuations in liquid water obtained in experiments and simulations.

## Model

We consider dynamics of longitudinal acoustic-like mode in liquid water on the length scale corresponding to the viscoelastic transition region in the framework of a mode-coupling theory (MCT) approach. Note that a model based on MCT reproduces correctly the experimental results of the time-resolved optical Kerr effect measurements of low-frequency vibrational dynamics in water over the temperature range of 247–353 K^11,82^. MCT can be formulated in the framework of a generalized hydrodynamic approach^83,84^. Several generalized hydrodynamic models have been developed. They are typically based on retaining the formal structure of the classical hydrodynamic theory and replacing macroscopic thermodynamic and transport coefficients with appropriate wave number-dependent memory functions. We follow the method proposed by Zwanzig et al.^85^.

 Based on the data of Liao et al. shown in Fig. 5 of Ref.^86^, we may approximately consider the heat mode to be decoupled from the sound mode for the parameters under study. Thus we neglect thermal relaxation and consider an isothermal case.

 The dynamic structure factor of the system can be defined as^87^

$$S({\mathbf{q}},\omega ) = \frac{1}{{2\rho_{0}^{{}} m}}\int\limits_{ - \infty }^{ + \infty } {dte^{ - i\omega t} \left\langle {\delta \rho ( - {\mathbf{q}},0)\delta \rho ({\mathbf{q}},t)} \right\rangle } ,$$

where $\delta \rho ({\mathbf{q}},t) = \int\limits_{{}}^{{}} {d{\mathbf{r}}e^{{i{\mathbf{q}} \cdot {\mathbf{r}}}} \delta \rho ({\mathbf{r}},t)}$ is the Fourier transform of the mass density fluctuation from the equilibrium value *ρ*_0_, **q** is the wave vector, $\omega$ is the angular frequency, $t$ is the time, $m$ is the molecular mass, and the bracket <> implies a thermal ensemble average.

 In the linear approximation, the generalized hydrodynamic equations for an isotropic system are reduced to the dynamic structure factor determined by the relation^87^

1 $$\frac{S(q,\omega )}{{S(q)}} = \frac{{2\Gamma (q)\omega_{0}^{2} (q)}}{{\left( {\omega^{2} - \omega_{0}^{2} (q)} \right)^{2} + 4\Gamma^{2} (q)\omega^{2} }}.$$

Here $\omega_{0}^{{}} (q)$ is the excitation frequency, $\Gamma (q)$ is the damping factor, $S(q)$ is the static structure factor, $q = \left| {\mathbf{q}} \right|$;

$$\omega_{0}^{2} (q) = \frac{{k_{B} Tq^{2} }}{mS(q)},$$

with $k_{B}$ and $T$ being the Boltzmann constant and the absolute temperature, respectively.

 Due to the viscoelastic transition, on increasing $q$, $\omega_{0}^{{}} (q)$ gradually bends upward from the linear adiabatic sound dispersion to the infinite frequency dispersion^75–81^. The onset of a substantial positive sound dispersion induced by the viscoelasticity accompanies also the decrease of temperature under supercooling conditions^80,88^. Accordingly, we use the following approximation for $\omega_{0}^{2} (q)$ up to wave numbers corresponding to the upper boundary of the viscoelastic transition region $q_{u}^{{}}$:

2 $$\omega_{0}^{2} (q) \approx k_{1} q^{2} + k_{2} q^{4} ,$$

with the coefficients $k_{1}^{{}}$ and $k_{2}^{{}}$ being constant. It should be emphasized that the approximation given by Eq. (2) is valid only for wave numbers less than ~ $q_{u}^{{}}$; this equation does not have a physical meaning for larger $q$. We estimate the values of $k_{1}$ and $k_{2}$ from a least-squares fit to the dispersion curves for $\omega_{0}$ obtained in inelastic X-ray scattering experiments^81^ for water at $\approx$ 278 K (case I) and in MD simulations for SPC/E model^89^ for D_2_O molecules at the temperature of maximum density for the SPC/E potential (case II). We get

2a $$k_{1} \approx {3}.{9}\times 10^{{6}} \;{\text{m}}^{{2}} /{\text{s}}^{{2}} ,\;k_{2} \approx {4}.{8}\times 10^{{ - {13}}} \;{\text{m}}^{{4}} /{\text{s}}^{{2}} \;\left( {\text{case I}} \right)$$

and

2b $$k_{1} \approx {3}.{2}\times 10^{{6}} \;{\text{m}}^{{2}} /{\text{s}}^{{2}} ,\;k_{2} \approx {7}.{7}\times 10^{{ - {13}}} \; {\text{m}}^{{4}} /{\text{s}}^{{2}} \;\left( {{\text{case}}\;{\text{ II}}} \right)$$

from the positions of $\omega_{0} (q)$ shown, respectively, in Fig. 2 of Ref.^81^ for $q$ up to $q_{u}^{{}}$ $\approx$ 3.0 nm^−1^ and in Fig. 2a of Ref.^89^ for $q$ up to $q_{u}^{{}}$ $\approx$ 3.1 nm^−1^. Note that we also complemented the data of Ref.^81^ by those from the inelastic ultraviolet scattering and Brillouin light scattering measurements^90^ for the wave number region from 0.02 to 0.1 nm^−1^; however, this did not noticeably change the values of $k_{1}$ and $k_{2}$.

 One can see that Eq. (1) with $\omega_{0}^{2} (q)$ given by Eq. (2) and $\Gamma (q)$ given by Eq. (3),

3 $$\Gamma (q) = \frac{{\eta_{L} }}{{2\rho_{0} }}q^{2} ,$$

with $\eta_{L}^{{}}$ being the generalized longitudinal viscosity, corresponds to a linearized version of the following MCT model:

4 $$\frac{{\partial \rho^{{}} }}{\partial t} + \nabla \cdot (\rho {\mathbf{u}}) = 0,$$

5 $$\frac{{\partial {\mathbf{u}}}}{\partial t} + {\mathbf{u}} \cdot \nabla {\mathbf{u}} = - \nabla \frac{\delta F[\rho ]}{{\delta \rho }} + \frac{1}{\rho }\nabla \cdot S,$$

6 $$F[\rho ] = \frac{1}{{2\rho_{0} }}\int {\left[ {k_{1} \rho^{2} + k_{2} (\nabla \rho )^{2} } \right]d{\mathbf{r}}} ,$$

7a $$S = S^{(av)} + \delta S$$

7b $$S_{\alpha \beta }^{(av)} = \frac{1}{2}\sum\limits_{\mu v} {\left[ {\eta_{S} (\delta_{\alpha \mu } \delta_{\beta \nu } + \delta_{\alpha \nu } \delta_{\beta \mu } )\left. { + (\eta_{L} - 2\eta_{S} )\delta_{\alpha \beta } \delta_{\mu \nu } } \right]} \right.} \left( {\frac{{\partial u_{\mu } }}{{\partial x_{\nu } }} + \frac{{\partial u_{\nu } }}{{\partial x_{\mu } }}} \right),$$

$$\left\langle {\delta S_{\alpha \beta } ({\mathbf{r}},t)} \right\rangle = 0,$$

$$\left\langle {\delta S_{\alpha \beta } ({\mathbf{r}}_{1} ,t_{1} )^{*} \delta S_{\mu \nu } ({\mathbf{r}}_{2} ,t_{2} )} \right\rangle =$$

7c $$= 2k_{B} T\left[ {\eta_{S} (\delta_{\alpha \mu } \delta_{\beta \nu } + \delta_{\alpha \nu } \delta_{\beta \mu } ) + (\eta_{L} - 2\eta_{S} )\delta_{\alpha \beta } \delta_{\mu \nu } } \right]\delta (t_{1} - t_{2} )\delta^{3} ({\mathbf{r}}_{1} - {\mathbf{r}}_{2} ).$$

Here ${\mathbf{r}} = (x_{1} ,x_{2} ,x_{3} )$ are the rectangular Cartesian coordinates, ${\mathbf{u}} = (u_{1} ,u_{2} ,u_{3} )$ is the velocity vector, $F[\rho ]$ is the free energy of the system, $\delta F[\rho ]/\delta \rho$ is the functional derivative of $F[\rho ]$ with respect to the density $\rho$ (the same symbols are used for the functional derivative and for the density fluctuation $\delta \rho$ which can lead to a confusion, thus we must conclude from the context which quantity is meant), $S$ is the deviatoric stress tensor that is represented as a sum of the mean and fluctuating components, $\eta_{L}^{{}}$ and $\eta_{S}^{{}}$ describe, respectively, the response of longitudinal and shear stresses to a change in the strain rate, $\delta_{\alpha \beta }$ is the Kronecker delta, $\delta (t)$ is the delta function, and the asterisk denotes complex conjugation. Equations (4)–(7) are treated as the equations of motion of hydrodynamic variables^84^.

 Assuming that the relative amplitudes of the density fluctuations are small but finite, we set for the finite amplitude density fluctuations in the first approximation, instead of Eq. (6):

8 $$F[\rho ] = \frac{1}{{2\rho_{0} }}\int {\left[ {k_{1} \delta \rho^{2} + k_{2} (\nabla \delta \rho )^{2} + \frac{\alpha }{3}\delta \rho^{3} } \right]d{\mathbf{r}}} ,$$

where $\alpha$ is a constant. Evidently, the coefficient $\alpha$ in Eq. (8) corresponds to the second derivative of pressure with respect to density, and thus to^91,92^

9 $$\frac{{\partial^{2} p}}{{\partial \rho^{2} }} = \frac{{2c^{2} (\beta - 1)}}{{\rho_{0} }},$$

where $\beta$ is the acoustic nonlinear coefficient and $c$ the speed of sound. For water at room temperature for low frequency^92^ $\beta$ $\approx$ 3.5. To obtain estimates for $\alpha$, we first estimate the apparent sound velocity, i.e., $c(q) = \omega_{0} (q)/q$, in the following way. The center of the viscoelastic crossover corresponds to the inflection point of the sound dispersion curve. Accordingly, for the dispersion curves given in Refs.^81,89^, the characteristic wave number for the region where the viscoelastic transition predominantly takes place is chosen to be $q^{*}$ = 2.0 nm^−1^ (case I) and $q^{*}$ = 2.5 nm^−1^ (case II). Then we get, respectively, $\omega_{0} (q^{*} )$ $\approx$ 4.9 ps^−1^ and $\omega_{0} (q^{*} )$ $\approx$ 6.8 ps^−1^, and consequently, $c(q^{*} )$ $\approx$ 2.5 $\times$ 10^3^ m/s and $c(q^{*} )$ $\approx$ 2.7 $\times$ 10^3^ m/s. Then, assuming that $\beta$ $\approx$ 3.5 and using Eq. (9), we have the following estimates

$$\alpha \approx {3}.0 \times {1}0^{{4}} \;{\text{m}}^{{5}} /{\text{kg}}\;{\text{s}}^{{2}} ,$$

10 $$\alpha \approx {3}.{7} \times {1}0^{{4}} \;{\text{m}}^{{5}} /{\text{kg}}\;{\text{s}}^{{2}} ,$$

in the case I and II, respectively. Note that the viscoelastic transition signifies a transformation from the low-frequency liquid-like behavior to the high-frequency solid-like behavior, while typically $\beta$ is higher for solids than for liquids^92^. Thus the above estimates for $\alpha$ may be considered as the estimates from below.

 In order to estimate the characteristic magnitude of density fluctuation, we note that the root mean square deviation of the number of molecules $N$ in a specified volume $V$, expressed as a fraction of the average number of molecules in the volume, is determined by^21^

11 $$\frac{{\left[ {\left\langle {\left( {N - \left\langle N \right\rangle } \right)^{2} } \right\rangle } \right]^{1/2} }}{\left\langle N \right\rangle } = \left( {\frac{{k_{B} T\kappa_{T} }}{V}} \right)^{1/2} ,$$

where $\kappa_{T}$ is the isothermal compressibility. The characteristic magnitude $A^{*}$ of density fluctuation $\delta \rho$ can be determined by this relation for a volume of water of size 1 nm^3^. (Note that the length scale of 1 nm is, probably, the smallest that can be examined outside the range of the local structure around individual molecules^21^.) We thus estimate $A^{*}$ $\approx$ 43 kg/m^3^ for water at $\approx$ 277 K ($\rho_{0}^{{}}$ $\approx$ 1.0 $\times$ 10^3^ kg/m^3^).

 Assuming that $\nabla \sim q^{*}$ and $\partial /\partial t\sim \omega_{0} (q^{*} )$, Eq. (4) gives for the magnitudes of the velocity components: $u_{\alpha }^{{}} \sim A^{*} \omega_{0} (q^{*} )/\rho_{0} q^{*}$. As $\delta F[\rho ]/\delta \rho \sim A^{*} \omega_{0} (q^{*} )^{2} /\rho_{0} q^{*2}$, the ratio of the term ${\mathbf{u}} \cdot \nabla {\mathbf{u}}$ to the term $\nabla (\delta F[\rho ]/\delta \rho )$ in Eq. (5) will be ~ $A^{*} /\rho_{0}$, a small quantity. Neglecting the relatively small nonlinear terms in Eqs. (4) and (5), one gets to leading order

$$\frac{\partial \delta \rho }{{\partial t}} + \rho_{0} \nabla \cdot {\mathbf{u}} = 0,$$

12 $$\frac{{\partial {\mathbf{u}}}}{\partial t} = - \nabla \frac{\delta F[\rho ]}{{\delta \rho }} + \frac{1}{{\rho_{0} }}\nabla \cdot S.$$

Then one obtains from Eqs. (7), (8), and (12)

13 $$\frac{{\partial^{2} \delta \rho }}{{\partial t^{2} }} - k_{1} \nabla^{2} \delta \rho + k_{2} \nabla^{4} \delta \rho - \alpha \nabla \cdot \left( {\delta \rho \nabla \delta \rho } \right) - \frac{{\eta_{L}^{{}} }}{{\rho_{0}^{{}} }}\nabla^{2} \frac{\partial \delta \rho }{{\partial t}} + \nabla^{2} \delta S = 0,$$

where the last term represents the fluctuating forces and obeys Eq. (7c). In one-dimensional case, we have

14 $$\frac{{\partial^{2} \delta \rho }}{{\partial t^{2} }} - k_{1} \frac{{\partial^{2} \delta \rho }}{{\partial x^{2} }} + k_{2} \frac{{\partial^{4} \delta \rho }}{{\partial x^{4} }} - \alpha \frac{\partial }{\partial x}\left( {\delta \rho \frac{\partial \delta \rho }{{\partial x}}} \right) - \frac{{\eta_{L} }}{{\rho_{0} }}\frac{{\partial^{2} }}{{\partial x^{2} }}\frac{\partial \delta \rho }{{\partial t}} + \frac{{\partial^{2} \delta S}}{{\partial x^{2} }} = 0,$$

$$\left\langle {\delta S(x,t)} \right\rangle = 0,$$

14a $$\left\langle {\delta S(x_{1} ,t_{1} )^{*} \delta S(x_{2} ,t_{2} )} \right\rangle = 2k_{B} T\eta_{L} \delta (t_{1} - t_{2} )\delta (x_{1} - x_{2} ).$$

Taking into account Eq. (11), we get the dimensionless version of Eqs. (14) and (14a):

15 $$\frac{{\partial^{2} \delta \rho^{\prime}}}{{\partial t^{{\prime}{2}} }} - k^{\prime}_{1} \frac{{\partial^{2} \delta \rho^{\prime}}}{{\partial x^{{\prime}{2}} }} + k^{\prime}_{2} \frac{{\partial^{4} \delta \rho^{\prime}}}{{\partial x^{{\prime}{4}} }} - \alpha^{\prime}\frac{\partial }{{\partial x^{\prime}}}\left( {\delta \rho^{\prime}\frac{{\partial \delta \rho^{\prime}}}{{\partial x^{\prime}}}} \right) - \nu^{\prime}_{L} \frac{{\partial^{2} }}{{\partial x^{{\prime}{2}} }}\frac{{\partial \delta \rho^{\prime}}}{{\partial t^{\prime}}} + \frac{{\partial^{2} \delta S^{\prime}}}{{\partial x^{{\prime}{2}} }} = 0,$$

$$\left\langle {\delta S^{\prime}(x^{\prime},t^{\prime})} \right\rangle = 0,$$

15a $$\left\langle \delta S^{\prime}{(x^{\prime}_{1} ,{t}^{\prime}_{1} )}^{*} \delta S^{\prime}(x^{\prime}_{2} ,t^{\prime}_{2} ) \right\rangle = \nu^{\prime 2}_{{L}} \delta (t^{\prime}_{1} - t^{\prime}_{2} )\delta (x^{\prime}_{1} - x^{\prime}_{2} ),$$

where

$$x^{\prime} = q^{*} x,\;t^{\prime} = \omega_{0} (q^{*} )t,\;\delta \rho^{\prime} = \delta \rho {/}A^{*},$$

$$\delta S^{\prime} = q^{*2} \delta S/A^{*} \omega_{0} (q^{*} )^{2},$$

$$k^{\prime}_{1} = \frac{{q^{*2} }}{{\omega_{0} (q^{*} )^{2} }}k_{1} ,\;k^{\prime}_{2} = \frac{{q^{*4} }}{{\omega_{0} (q^{*} )^{2} }}k_{2},$$

$$\alpha^{\prime} = \frac{{A^{*} q^{*2} }}{{\omega_{0} (q^{*} )^{2} }}\alpha ,\;\nu^{\prime}_{L} = \frac{{2\Gamma (q^{*} )}}{{\omega_{0} (q^{*} )}}.$$

We have the following estimates of the dimensionless parameters:

 for the case I

$$k^{\prime}_{1} \approx 0.{65},\;k^{\prime}_{2} \approx 0.32,\;\alpha^{\prime} \approx 0.22$$

for the case II

$$k^{\prime}_{1} \approx 0.{44},\;k^{\prime}_{2} \approx 0.65,\;\alpha^{\prime} \approx 0.22.$$

As to the parameter $\nu^{\prime}_{L}$, according to the data of Sacchetti et al.^93^, the ratio $2\Gamma (q)/\omega_{0}^{{}} (q)$ is about 0.60 for $q$ from 2 to 7 nm^−1^ for bulk water at room temperature, so that we choose

$$\nu^{\prime}_{L} \approx 0.60.$$

## Results and discussion

It is reasonable to focus on the dynamics of density variations on the time interval 0.5–1 ps, as shown by the time scales relevant to the considered system, listed below. (i) The time of structural relaxation of about 0.5–0.6 ps^76^. (ii) The time scale of 0.5–1 ps for LDL-like species revealed in time-resolved optical Kerr effect measurements of the vibrational dynamics and relaxation processes in bulk water in the temperature range 293–247 K^11^. (iii) The time scale of 0.5 ps for subensembles of water molecules found by the three-dimensional infrared spectroscopy (the three-dimensional infrared spectroscopy measurements sensitive to three-point frequency fluctuation correlation functions^60^ indicate that ambient water contains distinct subensembles of molecules with different dynamics, which do not interconvert on a time scale of 0.5 ps or somewhat longer than 0.5 ps). (iv) The time scale of 0.5–5 ps for the LDL-like species (in the temperature interval 340–250 K) determined in the simulations^27^ under the assumption of the extreme initial values of the LSI. (v) The timescale of primarily 1–2 ps for the low- and high-density regions (< 0.9 or > 1.1 kg/m^3^) observed in MD simulations^8^. (vi) The subpicosecond and picosecond timescales for Voronoi voids suggested by the simulations^18^.

 In the context of our model, we note that it is well-known that the two factors we consider, the nonlinear dispersion and nonlinear amplitude effects, can provide soliton^94^ (cf. Ref.^95^). In the idealized limit case $\eta_{L}^{{}} = 0$, $\delta S \equiv 0$, Eq. (13) is known as the Boussinesq equation, a nonlinear wave equation that indeed possesses exact $n$-soliton solutions^96^. A one-soliton solution of the form^97^

16 $$\delta \rho = A{\text{sech}}^{2} \frac{x - vt}{\delta },$$

16a $$A = - 12k_{2} /\alpha \delta^{2},$$

16b $$v^{2} = k_{1} - 4k_{2} /\delta^{2}$$

$$\left( \delta^{2} \ge 4k_{2} /k_{1} \right)$$

corresponds to a relatively stable localized density pulse. Here the parameters $A$, $v$, and $\delta$ characterize, respectively, the amplitude, velocity, and width of a pulse. These parameters are connected with each other through Eqs. (16a) and (16b), and $\delta$ can be considered as a free parameter, given that the condition of reality for $v$ is fulfilled. In view of Eq. (16b), the propagation velocity will be less than the sound velocity and will decrease with decreasing $\delta$.

Under the condition

17a $$\delta^{2} = 4k_{2} /k_{1},$$

Equation (16) determines the exact solution of Eq. (14) in the case $\eta_{L}^{{}} \ne 0$, $\delta S \equiv 0$:

17b $$\delta \rho = A{\text{sech}}^{2} \frac{x}{\delta },$$

17c $$A = - 3k_{1} /\alpha ,$$

$$v = 0.$$

Formally, this solution requires the fulfillment of the condition $\delta S \equiv 0$. However, numerical simulations (see “Methods”) of the corresponding non-dimensional problem (Eqs. (15) and (15a)) with the indicated above values of the parameters showed that the contribution of the stochastic term $\partial^{2} \delta S/\partial x_{{}}^{2}$ into the profile of $\delta \rho$ is negligibly small on the time interval up to 1 ps, for the initial profile given by Eqs. (17). Thus the exact solution (17) can be considered as a good approximation to the solution of Eqs. (14) and (14a) on this time interval.

 This result suggests the existence of a stationary soliton-like mode in ambient water on the time scale of 1 ps relevant to the considered system. In order to test this hypothesis, we compare the values of the amplitude and length scale of a localized density variation, following from our model, with available experimental and numerical data. At the beginning, it should be emphasized that in the viscoelastic transition region the dynamics of density fluctuations are strongly coupled with the formation and break-up of the hydrogen-bond network^78^. Thus the considered soliton solution may be interpreted as a stationary nanosized region of rarefication associated with rearrangement of hydrogen-bond network at the peak of the pulse.

 From Eqs. (17a) and (17c), we obtain the following estimates for $A$, $\delta$, and the full width at half maximum (FWHM) of a pulse. In the case I: $A$ $\approx$ 3.9 $\times$ 10^2^ kg/m^3^, $\delta$ $\approx$ 0.70 nm, and the FWHM of a pulse is $\approx$ 1.2 nm. In the case II: $A$ $\approx$ 2.6 $\times$ 10^2^ kg/m^3^, $\delta$ $\approx$ 0.97 nm, and the FWHM of a pulse is $\approx$ 1.7 nm. As indicated above, one may expect that $\alpha$ would be somewhat higher than the value given by Eq. (10) and, consequently, the amplitude $A$ would be lower.

 The obtained values of the amplitude $A$ of the soliton density pulse turn out to be comparable with an expected difference between the densities of LDL-like and HDL-like species, which is of the order of 200–300 kg/m^3^ (Refs.^21,34–36^). It should be emphasized that these LDL-like and HDL-like species were numerically observed as extreme and rare fluctuations^27^, as discussed in the introductory part of the paper in detail. The considered density variations can also be treated as those induced by externally applied perturbations. For instance, starting structures classified by the LSI, seeding in the simulations of Camisasca et al.^27^, may be treated, in fact, as initial perturbations which produce LDL-like and HDL-like regions well-defined on nanometer length and picosecond time scales. Considering external perturbations, we note that nanometer-sized density variations with amplitudes of the order of 2–4 $\times$ 10^2^ kg/m^3^ could be induced by mechanical forces in the biological range. Indeed, for a surface of size ~ 1 nm^2^, the effective pressure $p$ induced by external force of the order of 2 $\times$ 10^–9^ N can be estimated as ~ 2 $\times$ 10^9^ Pa. Then from the relation $\partial p/\partial \rho = c^{2}$ with $c$ = 2.5 $\times$ 10^3^ m/s as the characteristic value of the apparent sound velocity $c(q^{*} )$ (see earlier), one could estimate $\delta \rho$ ~ 3.2 $\times$ 10^2^ kg/m^3^, in the very first approximation.

 We next compare the obtained values of the length scale with available experimental and numerical results. It can be seen that the length scale of 1–2 nm, suggested by the soliton solution given by Eqs. (17), is consistent with the following results. (i) The molecular arrangement around the LDL-like and HDL-like species, determined by the extreme values of the LSI, in the simulations^27^ is well-defined over time at the distances ~ 2.2 nm. (ii) X-ray scattering studies^1,16^ determined the correlation length $\xi$ of density fluctuations to be of 0.3–0.4 nm. This correlation length can be related to an average length scale of density fluctuations, assuming a spherical shape with the diameter ~ 4.5 $\xi$, which gives ~ 1.5 nm at 280 K^1,16^. It should be noted that in many cases density variations will have sizes both smaller and larger than the indicated average size^8,16^. (iii) The length scale of low- and high-density regions in ambient water identified using Voronoi voids was found to be 1–2 nm^19^.

We now examine the temperature dependence of the length scales of the considered density variations. It is known that the low-frequency sound velocity is significantly decreased with decreasing temperature^90,98^, while the high-frequency sound velocity is increased^99^. Therefore one can expect that the ratio $k_{2} /k_{1}$ (and, consequently, $\delta$) will be increased with lowering temperature. Note that the average correlation length of density fluctuations in water is also increased with decreasing temperature^16^. In the case of supercooled water at 250 K (case III), one has the following data: $c(q)$ $\approx$ 1.3 $\times$ 10^3^ m/s at $q$ = 0.023 nm^-1^ (Ref.^90^) and $c(q)$ $\approx$ 3.5 $\times$ 10^3^ m/s at $q$ = $q_{u}^{{}}$ = 3 nm^-1^ (Ref.^99^). Then one can estimate $\omega_{0} (q)$ using the relation $\omega_{0} (q) = c(q)q$. In order to estimate $k_{1}$ and $k_{2}$ from these data, we first note that for the case I (the data of Refs.^81,90^), a least-squares fit to the dispersion curve for $\omega_{0}$ with only two reference points, $q$ = 0.023 nm^−1^ and $q = q_{u}^{{}} =$ 3.0 nm^−1^, gives $k_{1}$ $\approx$ 1.8 $\times$ 10^6^ m^2^/s^2^ and $k_{2}$ $\approx$ 7.3 $\times$ 10^–13^ m^4^/s^2^. These values are comparable to those obtained in Eq. (2a). Therefore we use this fit with the indicated above two reference points to estimate the values of $k_{1}$ and $k_{2}$ for the cases I and III by one and the same procedure. Then we obtain, in the very first approximation, $k_{1}$ $\approx$ 1.8 $\times$ 10^6^ m^2^/s^2^, $k_{2}$ $\approx$ 1.2 $\times$ 10^–12^ m^4^/s^2^, and $\delta$ $\approx$ 1.6 nm for supercooled water at 250 K (case III), and $\delta$ $\approx$ 1.3 nm for water at 278 K (case I). The ratio of these values of $\delta$, approximately equal to 1.3, is consistent with the ratio of the average correlation lengths of density fluctuations for 250 K and 277 K ($\approx$ 1.2)^16^. Thus it seems that the temperature-dependent length scales for density fluctuations in water and the stationary soliton solution to our model are in agreement with each other.

 The soliton ansatz (16) suggests the existence of propagating nonlinear eigenmodes of the system. In order to consider this possibility, we choose^21^ $A$/$\rho_{0}^{{}}$ = 0.043, in accordance with the estimation based on the root mean square deviation of the number of molecules in a volume of water of size 1 nm^3^. This corresponds to the characteristic amplitude of thermal fluctuations. Then from Eqs. (16a) and (16b), we have the following estimates: $\delta$ $\approx$ 2.1 nm (the FWHM of a pulse is $\approx$ 3.7 nm) and $v$ $\approx$ 1.9 $\times$ 10^3^ m/s in the case I; $\delta$ $\approx$ 2.4 nm (the FWHM of a pulse is $\approx$ 4.2 nm) and $v$ $\approx$ 1.6 $\times$ 10^3^ m/s in the case II. Our numerical simulations showed that on the time scale of 0.5 ps indicated in the beginning of this section, the exact soliton solution (16) can be considered as an approximation to the numerical solution of Eqs. (14) and (14a).

 We now consider the case of biomolecules hydration water. Within the low-wave number region $q \le$ 4 nm^−1^, the damping factors for hydration water of DNA and some proteins, such as the ribonuclease protein, are less than those for bulk water (in contrast to the high-wave number region)^68,100^. For example, for coherent density fluctuations propagating through DNA hydration water, $2\Gamma (q)/\omega_{0}^{{}} (q)$ is about 0.38 for $q$ = 3 nm^−1^ and 0.32 for $q$ = 2 nm^−1^, at the temperature 300 K and at the hydration level corresponding to DNA molecules surrounded by approximately two hydration shells^68^. In this case, since approximately $\omega_{0}^{{}} (q) \propto q$ and $\Gamma (q) \propto q^{2}$ (Refs.^68,100^), one can anticipate that the ratio $\Gamma (q)/\omega_{0}^{{}} (q)$ will be further decreased with decreasing $q$. Note that both the dispersion curves and the damping factors of the ribonuclease protein and DNA hydration water are very similar to each other in this wave number region^68,101^. Thus one can assume for this characteristic case

$$\nu^{\prime}_{L} \approx 0.32.$$

As a prototypical case of DNA/protein hydration water, we consider the case when $\nu^{\prime}_{L}$ = 0.32 and other parameters remain the same as earlier. Our numerical simulations showed that the exact solution (16) can be regarded as an approximation to the numerical solution of Eqs. (14) and (14a) on approximately two times longer time scale in comparison with the case of bulk water ($\nu^{\prime}_{L}$ = 0.60).

 We note that for hydration water of DNA and proteins, the high-frequency sound velocity is typically larger than that for bulk water^68,100^. For example, the propagation velocity of density fluctuations within DNA hydration water is of about 3500 m/s, which is significantly higher than for bulk water (3040 m/s)^68^. In this case, the ratio $k_{2} /k_{1}$ would be somewhat higher, and consequently, $\delta$ would be larger.

 To summarize, the results obtained predict the existence of stable, nearly stationary LDL-like states in ambient and supercooled water on the nanometer length scale and on the time scale up to ~ 0.5–1 ps. These states are associated with nonlinear eigenmodes of the system. Thus, the fully dynamic mechanism coming from the combination of the structural relaxation process and the finiteness of the amplitudes of terahertz longitudinal acoustic-like modes could contribute to the formation and dynamics of fluctuating nanosized density heterogeneities. The obtained results also suggest that for smaller amplitudes of density variations, determined by the average isothermal compressibility^21^, a perturbed soliton-like regime is also possible in liquid water on the time scale of ~ 0.5–1 ps.

 The soliton-like collective modes proposed by the present analysis may be connected, in particular, with dynamical heterogeneity in supercooled water. Indeed, spatially separated, extended low-mobility low-density regions and high-mobility high-density regions were observed in supercooled water in recent simulations^61,62^. Subsequent simulations^67^ revealed that ice nucleation occurs in the low-density regions. Thus one can suggest that the soliton-like collective excitations might be associated with rare, collective rearrangements in supercooled water from which ice is born^67,102^. The considered soliton-like collective modes can also be expected to be associated with some ultrafast biological processes such as those involving the energy transfer. For instance, they may be connected with protein-induced long-range perturbation of water dynamics extending up to ~ 2 nm or more from the protein surface^64,69^, discussed in the introductory part of the paper. Such long-range perturbation of the solvent’s collective dynamics was shown to be able to assist^64,103,104^ and even provide a necessary condition for activity of some proteins^103^. The dynamical heterogeneous distribution of water molecules in the vicinity of the interface of a biomolecule may also be important in such biological processes as protein-DNA binding which may occur in positions of local peaks of water density^68,105,106^. Thus, the mechanism suggested by the present analysis may support the corresponding long-range interactions^71,107^.

## Methods

For numerical simulations, we rewrite Eq. (15) as two first-order equations:

$$\frac{{\partial \delta \rho^{\prime}}}{{\partial t^{\prime}}} + \frac{{\partial u^{\prime}}}{{\partial x^{\prime}}} = 0,$$

18 $$\frac{{\partial u^{\prime}}}{{\partial t^{\prime}}} + \frac{{\partial g^{\prime}}}{{\partial x^{\prime}}} - h^{\prime} = 0,$$

where

$$g^{\prime} = k^{\prime}_{1} \delta \rho^{\prime} - k^{\prime}_{2} \frac{{\partial^{2} \delta \rho^{\prime}}}{{\partial x^{{\prime}{2}} }} + \frac{{\alpha^{\prime}}}{2}\left( {\delta \rho^{\prime}} \right)^{2} + \nu^{\prime}_{L} \frac{{\partial \delta \rho^{\prime}}}{{\partial t^{\prime}}},$$

and $h^{\prime}$ is related to the fluctuating force term $\partial^{2} \delta S^{\prime}/\partial x^{{\prime}{2}}$ in Eq. (15). Equations (18) with non-reflecting boundary conditions were solved numerically using a variant of the MacCormack method^108^ with spatial step $\Delta x^{\prime} =$ 10^–2^ and time step $\Delta t^{\prime} =$ 2.5 $\times$ 10^–5^. The length of the spatial lattice was chosen to be 120. We assume that at the grid node $x^{\prime}_{i}$, the following relations are satisfied:

$$\left\langle {h^{\prime}(x^{\prime}_{i} ,t^{\prime})} \right\rangle = 0,$$

$$\left\langle {h^{\prime}(x^{\prime}_{i} ,t^{\prime}_{1} )^{*} h^{\prime}(x^{\prime}_{i} ,t^{\prime}_{2} )} \right\rangle = \nu^{\prime 2}_{L} \delta (t^{\prime}_{1} - t^{\prime}_{2} ),$$

in consistency with Eq. (15a). The simulations showed numerical stability for the indicated values of the parameters.

## Data Availability

The datasets generated during and/or analyzed during the current study are available from the corresponding author on reasonable request.
